# CMAtlas: a comprehensive DNA methylation atlas for exploring epigenetic alterations in 34 human cancer types

**DOI:** 10.1093/bioinformatics/btag022

**Published:** 2026-01-14

**Authors:** Mengni Liu, Lizhen Jiang, Luowanyue Zhang, Tianjian Chen, Xingzhe Wang, Yuan Liang, Xianping Shi, Jian Ren, Yueyuan Zheng

**Affiliations:** Clinical Big Data Research Center, Scientific Research Center, Shenzhen Key Laboratory of Bone Tissue Repair and Translational Research, Department of Orthopaedic Surgery, The Seventh Affiliated Hospital of Sun Yat-sen University, Shenzhen 518107, China; State Key Laboratory of Oncology in South China, Cancer Center, Collaborative Innovation Center for Cancer Medicine, School of Life Sciences, Sun Yat-sen University, Guangzhou 510060, China; Clinical Big Data Research Center, Scientific Research Center, Shenzhen Key Laboratory of Bone Tissue Repair and Translational Research, Department of Orthopaedic Surgery, The Seventh Affiliated Hospital of Sun Yat-sen University, Shenzhen 518107, China; The Affiliated Traditional Chinese Medicine Hospital, Guangzhou Medical University, Guangzhou, Guangdong 510182, China; Sino-French Hoffmann Institute, Guangdong Provincial Key Laboratory of Protein Modification and Disease, School of Basic Medical Science, Guangzhou Medical University, Guangzhou, Guangdong 510182, China; State Key Laboratory of Oncology in South China, Cancer Center, Collaborative Innovation Center for Cancer Medicine, School of Life Sciences, Sun Yat-sen University, Guangzhou 510060, China; State Key Laboratory of Oncology in South China, Cancer Center, Collaborative Innovation Center for Cancer Medicine, School of Life Sciences, Sun Yat-sen University, Guangzhou 510060, China; Clinical Big Data Research Center, Scientific Research Center, Shenzhen Key Laboratory of Bone Tissue Repair and Translational Research, Department of Orthopaedic Surgery, The Seventh Affiliated Hospital of Sun Yat-sen University, Shenzhen 518107, China; Clinical Big Data Research Center, Scientific Research Center, Shenzhen Key Laboratory of Bone Tissue Repair and Translational Research, Department of Orthopaedic Surgery, The Seventh Affiliated Hospital of Sun Yat-sen University, Shenzhen 518107, China; The Affiliated Traditional Chinese Medicine Hospital, Guangzhou Medical University, Guangzhou, Guangdong 510182, China; Sino-French Hoffmann Institute, Guangdong Provincial Key Laboratory of Protein Modification and Disease, School of Basic Medical Science, Guangzhou Medical University, Guangzhou, Guangdong 510182, China; State Key Laboratory of Oncology in South China, Cancer Center, Collaborative Innovation Center for Cancer Medicine, School of Life Sciences, Sun Yat-sen University, Guangzhou 510060, China; Clinical Big Data Research Center, Scientific Research Center, Shenzhen Key Laboratory of Bone Tissue Repair and Translational Research, Department of Orthopaedic Surgery, The Seventh Affiliated Hospital of Sun Yat-sen University, Shenzhen 518107, China

## Abstract

**Motivation:**

Aberrant DNA methylation is a fundamental epigenetic hallmark of cancer. However, existing resources often lack technological diversity and comprehensive cancer coverage. Furthermore, most platforms fail to achieve deep multi-omics integration and tend to ignore cancer-type-specific methylation features, limiting their utility in precision oncology and drug discovery.

**Results:**

We developed Cancer Methylation Atlas (CMAtlas), a comprehensive platform integrating 13 753 samples across 34 cancer types. By applying technology-tailored pipelines to data from various profiling technologies, we identified 830 725 tumor-specific differentially methylated elements (DMEs) and 1 480 098 differentially methylated regions (DMRs), alongside 1 154 256 cancer-type-specific DMEs and 329 154 DMRs. The platform demonstrates high cross-platform consistency and strong concordance between tumor tissues and cell lines, ensuring the robustness of our findings. All DMEs and DMRs are annotated with multi-omics data (RNA expression, somatic mutations, and chromatin accessibility) and clinical relevance (survival associations and cell-free DNA profiling). We further demonstrate the utility of CMAtlas by identifying prognostic aberrant methylation in colorectal cancer driver genes.

**Availability and implementation:**

CMAtlas is freely accessible at {{https://cmatlas.renlab.cn/}}. The platform offers an intuitive web interface supporting gene-centric and cancer-centric queries, alongside customizable analysis modules designed to facilitate user-specific research needs.

## 1 Introduction

Aberrant DNA methylation is now widely recognized as a hallmark of cancer, intricately linked to oncogenesis, tumor progression, and therapeutic response (Jones and Baylin 2002, Koch *et al.* 2018). DNA methylation alterations occur in diverse genomic contexts, including promoters, enhancers and gene bodies, as well as in regions with varying CpG densities such as CpG islands, shores and shelves. These alterations contribute significantly to tumorigenesis by silencing tumor suppressor genes, activating oncogenes, and promoting genomic instability (Berman *et al.* 2011, Lakshminarasimhan and Liang 2016, Sheaffer *et al.* 2016). Generally, cancer genomes often exhibit global DNA hypomethylation compared to normal cells (Berdasco and Esteller 2019). At the same time, localized hypermethylation, particularly within CpG islands (CGI) in gene promoter regions, frequently precipitates the silencing of tumor suppressor genes (TSGs) (Berdasco and Esteller 2019). Moreover, dysregulation of DNA methylation at enhancer regions, critical cis-regulatory sequences modulating gene transcription, can disrupt normal gene expression programs and promote malignant transformation (Aran and Hellman 2013). Accumulating evidence also highlights the existence of cancer-type-specific DNA methylation pattern, reflecting the diverse etiologies and molecular landscapes of distinct malignancies (Irizarry *et al.* 2009, Witte *et al.* 2014). Consequently, a comprehensive investigation of both tumor-specific and cancer-type-specific methylation aberrations is essential for deciphering the epigenetic basis of tumorigenesis and advancing precision oncology.

Recent advances in methylation profiling technologies, encompassing both bulk-level [e.g. Illumina DNA methylation array, reduced representation bisulfite sequencing (RRBS), whole genome bisulfite sequencing (WGBS), and single-cell DNA methylation approaches (scMeth-seq, including scBS-seq and scTrioSeq2)], have facilitated the generation of large-scale DNA methylomes across numerous cancer types. Leveraging the varying resolution and genomic breadth offered by these profiling technologies is crucial, as their complementary nature can facilitate robust cross-validation and a more nuanced understanding of methylation alterations across different genomic contexts. While existing cancer-associated methylation databases (Ding *et al.* 2020, Huang *et al.* 2021, Xing *et al.* 2022, Xiong *et al.* 2022, Zong *et al.* 2022, Zhang *et al.* 2023, Zhu *et al.* 2024) are valuable resources for studying aberrant methylation, they commonly exhibit several critical limitations: (i) primarily rely on a single profiling technology (e.g. MethMarkerDB and MethBank4.0 for WGBS; EWAS Open Platform, MethHC2.0 and DNMIVD for arrays) (Ding *et al.* 2020, Huang *et al.* 2021, Xiong *et al.* 2022, Zhang *et al.* 2023, Zhu *et al.* 2024); (ii) typically offer limited coverage of cancer types (e.g. MethBank4.0, MethMarkerDB typically cover ≤15 cancer types) and generally lack cancer cell line data (Zhang *et al.* 2023, Zhu *et al.* 2024); (iii) insufficient integration of multi-omics data and clinical annotations (Ding *et al.* 2020, Xing *et al.* 2022, Xiong *et al.* 2022, Zhang *et al.* 2023), limiting their utility in mechanistic and translational studies; (iv) primarily focus on tumor-specific methylation alterations within individual cancers, often overlooking cancer-type-specific epigenetic features.

**Figure 1:**
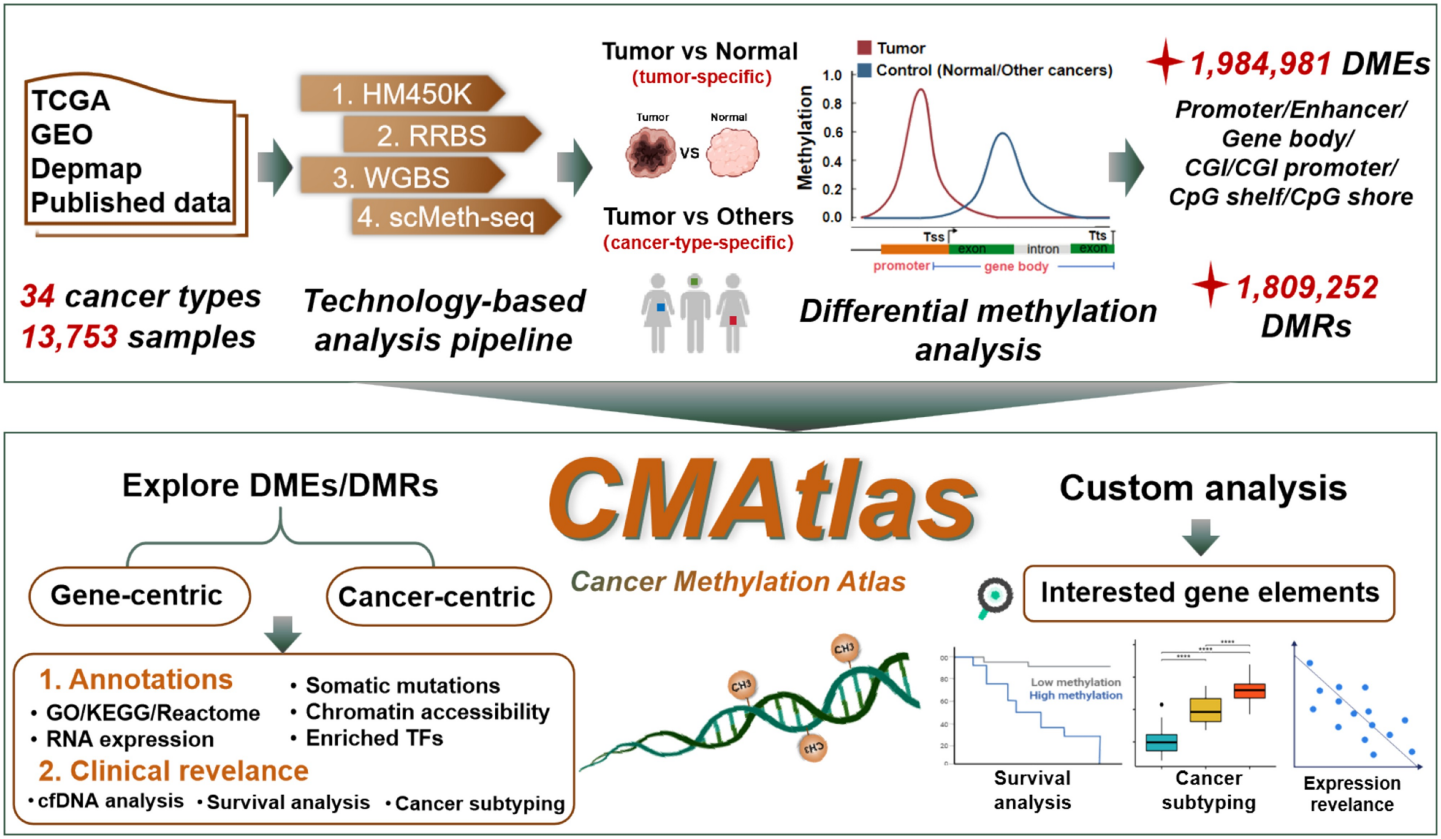
Overall design and construction of CMAtlas. Methylation data generated by HM450K, RRBS, WGBS and scMeth-seq across 34 cancer types were collected from TCGA, GEO, DepMap, and published datasets. CMAtlas offers comprehensive multi-omics annotaions for DMEs and DMRs, along with custom analysis modules that enables users to explore gene elements of interest.

To address these critical gaps, we present Cancer Methylation Atlas (CMAtlas, https://cmatlas.renlab.cn/), a comprehensive pan-cancer resource built upon extensive collection and rigorous processing of multi-technology methylation data using technology-tailored pipelines. CMAtlas incoperates 13 753 tissue and 676 cancer cell line samples across 34 cancer types derived from TCGA, GEO, DepMap, and manually curated datasets (Fig. 1). The platform systematically identifies tumor-specific and specifically, cancer-type-specific differentially methylated regions (DMRs) and elements (DMEs), thereby providing distinct perspectives on epigenetic dysregulation. DMEs focus on methylation changes in defined functional units (e.g. promoters, enhancers, or CpG islands) for locus-specific insights, whereas DMRs delineate contiguous epigenetic regions at various scales to reveal regional regulatory shifts. Furthermore, all identified DMEs and DMRs are annotated with multi-dimensional features: (i) functional characterization via gene set enrichment and transcription factor (TF) binding motif analysis; (ii) multi-omics integration incorporating RNA expression, somatic mutations, and chromatin accessibility data; (iii) clinical relevance including survival associations, cancer molecular subtypes, and non-invasive biomarker potential via cell-free DNA (cfDNA) profiling. By offering an intuitive web interface that supports both gene- and cancer-centric exploration, along with customizable analysis modules, CMAtlas serves as a biologically interpretable and clinically actionable resource for dissecting context-specific epigenetic dysregulation and advancing precision oncology.

## 2 Materials and methods

### 2.1 Data collection

CMAtlas incorporates high-quality methylation data generated using multiple technologies. Specifically, HM450k methylation data were downloaded from TCGA using TCGABiolinks package (version 2.30.4) (Colaprico *et al.* 2016) and from GEO database. RRBS sequencing data were obtained from GEO and SRA databases, while tumor cell line RRBS data were downloaded from DepMap project (Ghandi *et al.* 2019). WGBS data were acquired from GEO, SRA, and previously published datasets (Zhou *et al.* 2018). scMeth-seq data were collected from GEO and scMethBank (Zong *et al.* 2022). To further enhance the diagnostic strategy, we systematically curated 588 cfDNA methylation samples from GEO, SRA, and GSA databases across six distinct cancer types.

In addition to the methylation data, CMAtlas integrates a wealth of multi-omics data, including: (i) RNA expression data of tumor and normal tissues obtained from TCGA and GTEX project, as well as data of tumor cell lines from DepMap; (ii) somatic mutations data of tumor tissues and tumor cell lines acquired from TCGA and DepMap separately; (iii) ATAC-seq bigWig data obtained from TCGA; and (iv) clinical information from TCGA, including survival data and cancer molecular subtype information.

### 2.2 DMEs identification

Annotatr (version 1.28.0) (Cavalcante and Sartor 2017) was used to annotate regulatory elements based on GENCODE gene annotation file (v48). Specifically, CGI promoters were identified as those overlapping with CGIs. CGI shores were defined as regions up to 2 kb away from CGIs, and CGI shelves as regions located 2–4 kb from the CGIs. Enhancer regions were retrieved from the GeneHancer database (Fishilevich *et al.* 2017). DMEs were identified according to the following criteria:

- contain at least three CpG sites;
- be covered in ≥ 30% of samples within each comparison group;
- show an absolute mean methylation difference ≥ 0.1 in each comparative analysis;
- exhibit statistically significant differences (false discovery rate [FDR] < 0.05, assessed by a Student’s *t*-test with Benjamini–Hochberg correction).

### 2.3 DMRs identification

To identify DMRs in each cancer type, we performed a genome-wide screening using metilene (version 0.2–8) (Juhling *et al.* 2016) with the following parameters: a minimum of 10 CpGs per DMR (-m 10), a mean absolute methylation difference of at least 0.1 (-d 0.1), and a minimum of 30% samples support in each comparison group (-X 0.3 -Y 0.3). DMRs were considered significant based on a FDR threshold of < 0.05. Genomic annotation of the identified DMRs was performed with ChIPseeker (version 1.38.0) (Yu *et al.* 2015) using the GENCODE gene annotation (release v48).

### 2.4 Database and web interface implementation

All data in CMAtlas were stored and managed using MySQL tables. The server-backend was developed based on Java, while the web-frontend interfaces were implemented using HyperText Markup Language (HTML), Cascading Style Sheets (CSS) and JavaScript (JS). All the interactive charts were generated by ECharts to visualize the analysis results.

Detailed methods are provided in the Supplementary Materials, available as supplementary data at *Bioinformatics* online.

## 3 Results

### 3.1 Database content

CMAtlas currently integrates a total of 13 753 samples across 34 cancer types, profiled using multiple high-throughput methylation sequencing technologies. Specifically, the database includes data from HM450K and RRBS (each covering 28 cancer types), WGBS (11 cancer types), and scMeth-seq (2 cancer types) (Table 1). COAD/READ, BRCA and PRAD comprise the largest sample cohort. The resource encompasses both tissue and cell line samples, including 11 325 tumor tissue samples, 1752 normal tissue samples and 676 tumor cell line samples.

**Table 1:** Numbers of normal and tumor samples for each cancer type obtained from different sequencing technologies.

Cancer type	Tissue								Cell line
	Array		RRBS		WGBS		scMeth-seq		RRBS
	Tumor	Normal	Tumor	Normal	Tumor	Normal	Tumor	Normal	Tumor
COAD/READ	600	337	82	10	–	–	992	87	53
BRCA	785	97	48	–	–	–	–	–	42
PRAD	592	112	–	–	15	4	81	61	5
LUAD	622	51	29	–	–	–	–	–	59
HNSC	548	61	16	16	4	4	–	–	31
KIRC	400	215	10	10	–	–	–	–	10
THCA	507	56	40	31	–	–	–	–	11
LGG	562	5	–	–	–	–	–	–	–
BLCA	458	67	–	–	–	–	–	–	22
SKCM	470	2	–	–	–	–	–	–	49
LIHC	417	70	4	2	4	4	–	–	17
STAD	414	21	47	–	–	–	–	–	31
UCEC	431	46	–	–	–	–	–	–	16
PAAD	370	54	11	2	–	–	–	–	33
LUSC	370	42	27	–	–	–	–	–	16
ESCC	129	32	43	–	73	56	–	–	22
CESC	307	3	44	–	–	–	–	–	–
KIRP	275	45	–	–	–	–	–	–	–
SARC	261	4	–	–	–	–	–	–	25
LAML	194	–	3	4	24	20	–	–	31
EAC	212	33	–	–	12	7	–	–	–
CHOL	174	13	11	–	–	–	–	–	–
GBM	141	2	–	–	6	17	–	–	27
OV	30	18	48	–	–	–	–	–	44
THYM	124	2	–	–	–	–	–	–	–
MESO	87	–	–	–	–	–	–	–	7
CLL	–	–	43	13	6	16	–	–	–
DLBC	48	–	–	–	–	–	–	–	17
UCS	57	–	–	–	–	–	–	–	–
ALL	–	–	–	–	7	16	–	–	25
SCLC	–	–	–	–	–	–	–	–	48
MM	–	–	–	–	5	16	–	–	21
MCL	–	–	–	–	5	16	–	–	–
LCML	–	–	–	–	–	–	–	–	14

**Figure 2:**
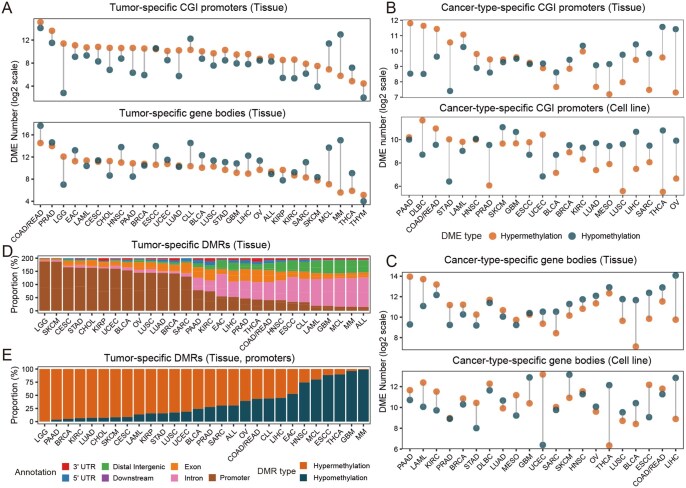
CMAtlas comprehensively characterizes both tumor-specific and cancer-type-specific DMEs and DMRs across human cancers. (A) Numbers of tumor-specific hypermethylated and hypomethylated CGI promoters and gene bodies across 29 cancer types. (B) Numbers of cancer-type-specific hypermethylated and hypomethylated CGI promoters and (C) gene bodies across 21 common cancer types, identified in both tumor tissues and tumor cell lines. (D) Genomic distribution of DMR annotations across 28 cancer types. (E) Proportions of hyper-/hypomethylated DMRs annotated as promoters regions using ChIPSeeker (Yu *et al.* 2015).

To systematically identify cancer-associated methylation alterations, CMAtlas used a rigorous analytical pipeline to detect tumor-specific DMEs and DMRs for each cancer type, as well as cancer-type-specific DMEs and DMRs. In total, we identified 830 725 tumor-specific DMEs and 1 480 098 DMRs, alongside 1 154 256 cancer-type-specific DMEs and 329 154 DMRs. Regarding tumor-specific DMEs (Fig. 2A), most cancer types (24/29) exhibited a higher proportion of hypermethylated CGI-promoters, a pattern consistent with the frequent silencing of TSGs observed across various cancers (Berdasco and Esteller 2019). Additionally, the majority of cancer types (19/29) displayed more hypomethylated gene bodies, aligning with the well-established global hypomethylation pattern characteristic of malignancies (Berdasco and Esteller 2019). Other DME types showed more heterogeneous patterns across cancer types. Notably, a high degree of concordance was observed between cancer-type-specific DMEs identified in tumor tissues and those from tumor cell lines, with 16/21 cancer types showing consistent methylation patterns in CGI promoters (Fig. 2B) and 15/21 cancer types showing consistent patterns in gene bodies (Fig. 2C), indicating strong consistency in methylation profiles between these two model systems. Examination of DMR distribution indicated that tumor-specific DMRs are predominantly located in promoter and intronic regions (Fig. 2D). In line with DME patterns, hypermethylated DMR-promoters exceeded hypomethylated ones in most cancers (21/28) (Fig. 2E). Taken together, these results demonstrate the robustness and accuracy of the CMAtlas analytical pipeline.

### 3.2 Web interface and usage

**Figure 3:**
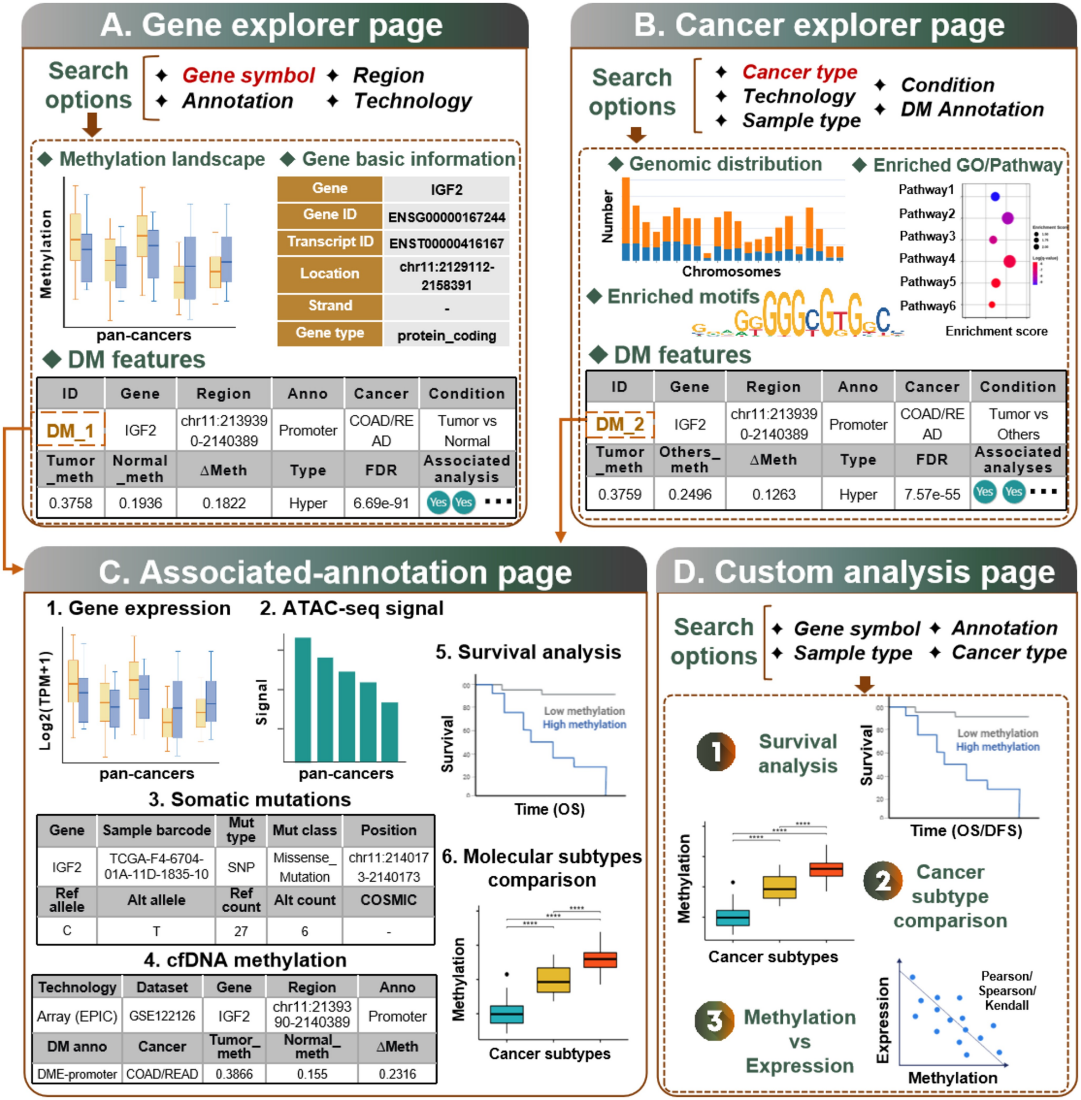
Overview of the CMAtlas web interface. (A) The Gene explorer interface of CMAtlas. (B) The Cancer explorer interface of CMAtlas. (C) Multi-omics annotations of DMEs and DMRs are accessible by clicking on the corresponding ID in the Gene Explorer and Cancer Explorer pages. (D) Custom Analysis Suite: Supports user-defined survival, subtype comparison, and methylation-expression correlation analyses with flexible parameterization.

CMAtlas provides a user-friendly web interface that enables researchers to explore differentially methylated (DM) features (both DMEs and DMRs) interactively across various cancer types (Fig. 3).

The Gene explorer module (Fig. 3A) enables gene-level investigation of DMEs through user-defined parameters (Gene symbol, Region, and Technology). This module displays basic gene information for the selected gene, followed by a visualization of pan-cancer methylation landscape for the specified genomic elements. Tabular summaries of DMEs comparing tumor versus normal and tumor versus other cancers are presented, where each entry links to a dedicated annotation subpage via a unique ID. Only statistically significant entries are displayed (|Δmethylation| ≥ 0.1, *t*-test with Benjamini-Hochberg correction, FDR < 0.05). The Cancer explorer module (Fig. 3B) supports exploration of DMEs and DMRs at the cancer-type level by selecting specific parameters including “Cancer type”, “Technology”, “Sample type” (tissue/cell line), and “Condition” (Tumor versus Normal/Tumor versus Others). Upon parameter selection, the chromosomal distribution of differential methylation features is displayed. Similar to the Gene explorer, detailed tables of DMEs and DMRs are presented. Additionally, functional enrichment analyses—including GO, KEGG, and Reactome pathways are provided for both hypermethylated and hypomethylated features, presented as dot plot visualizations and detailed tables.

TF binding motif enrichment results are also displayed in tables for hyper- and hypo-DM features.

The associated annotation modules (Fig. 3C) provide comprehensive multi-omics annotations for specific DM features. They are accessible by clicking the “ID” in either the “Gene explorer” or “Cancer explorer” pages. The annotations include: (i) gene expression landscape of the specific gene across pan-cancers; (ii) ATAC-seq signal of the corresponding regions across pan-cancers; (iii) somatic mutations located within the DM feature regions; (iv) comparison of cfDNA methylation patterns in corresponding regions between tumor and normal samples; (v) overall survival (OS) analysis based on TCGA clinical data; and (vi) comparative methylation analysis across cancer molecular subtypes.

Three custom analysis modules (Fig. 3D) offer additional functionality: survival analysis, cancer subtype comparisons, and methylation-expression correlation analysis. Users can specify parameters including “Gene symbol”, “Annotation”, “Sample type”, and “Cancer type”. For survival analysis, users can conduct analyses based on OS or disease-free survival (DFS). TCGA patients are stratified based on methylation levels using median, quartile cutoffs, or user-defined thresholds. The analysis also incorporates cancer molecular subtype information, enabling subtype-specific survival assessments. Additionally, users can compare methylation levels between different cancer molecular subtypes to identify potential subtype-specific biomarkers and investigate correlations between gene expression and methylation levels to elucidate regulatory epigenetic mechanisms.

CMAtlas provides comprehensive data accessibility through a dedicated “Download” page, where users can retrieve all tumor- and cancer-type-specific DMEs and DMRs. Detailed usage guidelines are available on the “Help” page. Overall, CMAtlas serves as a valuable resource for both panoramic exploration of methylation alterations and in-depth mechanistic investigations, satisfying diverse research needs in cancer epigenomics.

### 3.3 Leveraging CMAtlas to elucidate methylation regulatory mechanisms in COAD/READ

**Figure 4:**
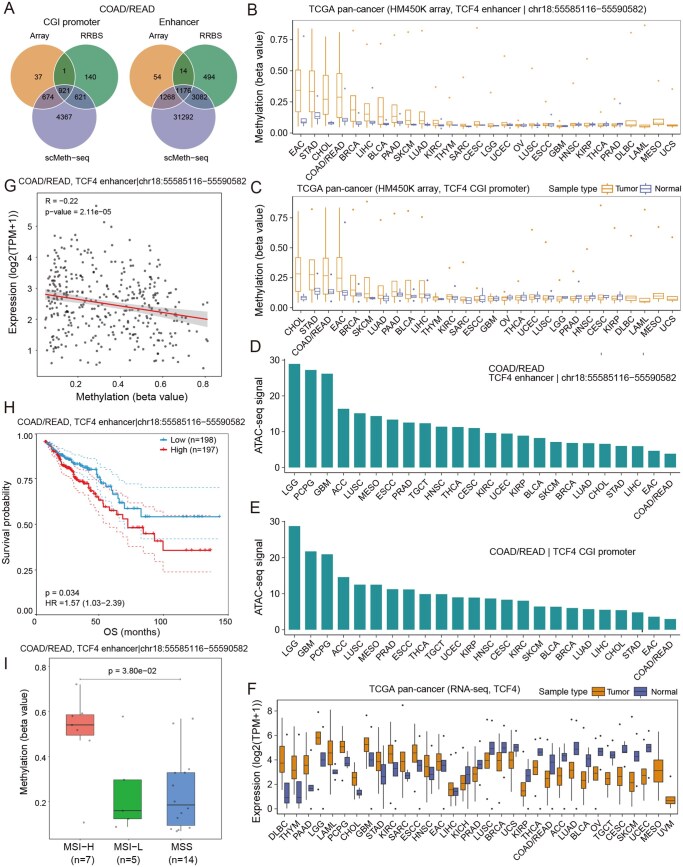
Application of CMAtlas in COAD/READ analysis. (A) Venn diagram illustrating the overlap of CGI promoters and enhancers identified by Array, RRBS, and scMeth-seq. (B and C) Pan-cancer DNA methylation profile from TCGA HM450K array for *TCF4* enhancer (B) and CGI promoter (C). (D and E) Pan-cancer chromatin accessibility profile from TCGA ATAC-seq for the hypermethylated *TCF4* enhancer (D) and CGI promoter (E). (F) Pan-cancer gene expression profile of *TCF4* based on TCGA RNA-seq data. (G) Correlation between methylation level of *TCF4* enhancer and *TCF4* expression using Pearson correlation. (H) Overall survival analysis of COAD/READ patients stratified by the median methylation value of the *TCF4* enhancer. (I) Comparison of COAD/READ molecular subtypes based on methylation levels of the *TCF4* enhancer.

To demonstrate the utility of CMAtlas in uncovering the methylation-mediated regulatory mechanisms in tumorigenesis, we focused on COAD/READ—the largest dataset in CMAtlas—as an illustrative example. A substantial number of DMEs were consistently identified across multiple sequencing platforms, with particularly high concordance between Array and RRBS data (Fig. 4A).

Given that aberrant DNA methylation can promote tumor development by silencing TSGs or activating oncogenes, thereby disrupting cellular homeostasis, we first interrogated CMAtlas for methylation patterns associated with the expression of the top candidate 500 oncogenes and 500 TSGs, as estimated by cancer mutation signatures curated in the TUSON database (Davoli *et al.* 2013).

Subsequent differential methylation analysis using CMAtlas revealed that 347 TSGs were hypermethylated either in CGI promoters or enhancers compared to normal tissues. Among these, 29 TSGs showed significantly reduced expression levels relative to normal controls, encompassing 84 hypermethylated enhancers and 11 hypermethylated CGI promoters. Notably, *TCF4*, a critical regulator of intestinal development and differentiation (van Es *et al.* 2012), harbored concurrent hypermethylation at both CGI promoters and enhancers, consistently detected by both Array and scMeth-seq techonologies (Fig. 4B and C, Fig. 1A and B, available as supplementary data at *Bioinformatics* online). While *TCF4* deficiency has been implicated in colorectal cancer liver metastasis (Tu *et al.* 2021), the underlying epigenetic mechanisms remain poorly understood. Using CMAtlas, we observed that these hypermethylated regions displayed markedly reduced chromatin accessibility compared to other cancer types (Fig. 4D and E). This finding leads us to hypothesize that the hypermethylation-induced local chromatin compaction at the *TCF4* locus might disrupt the binding of the basal transcription machinery at the regulatory regions, accounting for the profound loss of *TCF4* expression observed in colorectal cancer (Fig. 4F and G, Fig. 1C, available as supplementary data at *Bioinformatics* online). Clinically, these methylation alterations were significantly correlated with favorable patient outcomes and effectively distinguished molecular subtypes of COAD/READ (Fig. 4H and I, Fig. 1D, available as supplementary data at *Bioinformatics* online), underscoring their potential as prognostic biomarkers. Furthermore, a consistent hypermethylation pattern of *TCF4* enhancer was detected in cfDNA from COAD/READ patients, with mean methylation levels of 0.4526 in patient-derived cfDNA compared to 0.1358 in healthy individuals, indicating their utility for non-invasive early detection and disease monitoring. Importantly, this hypermethylation pattern of *TCF4* enhancer and promoter was uniquely observed in gastrointestinal (GI) cancers, including esophageal adenocarcinoma, stomach adenocarcinoma, cholangiocarcinoma and COAD/READ, suggesting a GI cancer-specific methylation signature.

Analysis of the TUSON oncogene set using CMAtlas identified 376 oncogenes with hypomethylation in CGI promoters or enhancers. Among these, 37 oncogenes showed significantly higher expression than normal tissues and were linked to 153 hypomethylated enhancers and 5 hypomethylated CGI promoters. Notably, a hypomethylated enhancer associated with *MGAT5* was reproducibly detected by both RRBS and scMeth-seq (Fig. 1E and F, available as supplementary data at *Bioinformatics* online). Although *MGAT5* has previously been implicated in colorectal cancer progression, the epigenetic basis of its regulation remains unclear (Petretti *et al.* 2000, Zhan *et al.* 2024). Remarkably, this DME exhibited the highest correlation with both gene expression level and chromatin accessibility across pan-cancers (Fig. 1G and H, available as supplementary data at *Bioinformatics* online), suggesting that demethylation facilitates TF binding to drive *MGAT5* overexpression and colorectal carcinogenesis.

Collectively, these findings highlight the value of CMAtlas in elucidating both tumor-specific and cancer-type-specific epigenetic mechanisms of tumorigenesis, as well as in identifying clinically actionable biomarkers for prognosis and non-invasive detection.

## 4 Summary and perspectives

In this study, we introduce CMAtlas, a comprehensive pan-cancer resource that integrates multi-dimensional DNA methylation data across 34 cancer types. Compared with existing resources, CMAtlas offers four key advantages (Table 2): (i) To our knowledge, CMAtlas is the first pan-cancer resource to jointly incorporate both bulk (HM450K, RRBS, WGBS) and single-cell methylation data. This multi-resolution framework enables both broad cross-platform validation and the bridging of population-level trends with single-cell epigenetic heterogeneity, while preserving the distinct strengths of each technology. (ii) CMAtlas encompasses a wider range of cancer types and sample types (tissue and cell line), offering a more thorough representation of cancer methylomes and enhancing its utility for experimental design. The high concordance between DMEs derived from tumor tissues and those from cell lines further validates the reliability of our resource (Fig. 2B and C); (iii) CMAtlas integrates extensive and biologically meaningful annotations, covering functional characterization (e.g. gene set enrichment and TF motif analysis), multi-omics integration (RNA expression, somatic mutations, and chromatin accessibility), and detailed clinical relevance (survival, subtypes, and cfDNA biomarker potential); (iv) While most existing resources primarily focus on tumor-specific methylation patterns, CMAtlas additionally provides a dedicated analysis and annotation of cancer-type-specific methylation features, which empowers precision oncology by revealing epigenetic drivers unique to specific cancer contexts.

**Table 2:** Comparison of cancer-associated methylation web servers.

Web server	CMAtlas	DiseaseMeth3.0	MethMarkerDB	MethBank4.0	EWAS Open Platform	MethHC2.0	DNMIVD	scMethBank
**Accessible**	Fully	Partially	Fully	Fully	Fully	Partially	Fully	Fully
**Technology**	Array, RRBS, WGBS, scMeth-seq	Array, RRBS, WGBS	WGBS	WGBS	Array	Array	Array	scMeth-seq
**Cancer type**	34	33	13	12	60	33	23	1
**Sample type**	tissue & cell line	tissue	tissue	tissue & cell line	tissue & cell line	tissue	tissue	tissue
**Feature types**	DME, DMR	DMG	DMR	DMR, DMG	DME	DME	DMG	
**Cancer-type-specific features**	√	×	×	×	×	×	×	×
**Enriched TFs**	√	×	√	√	√	×	×	×
**Associated mutations**	√	×	√	×	×	√	×	×
**Chromatin asscessibility**	√	×	√	×	×	×	×	×
**cfDNA/ctDNA**	cfDNA	×	×	×	×	ctDNA	×	×

To demonstrate the utility of CMAtlas, we conducted an in-depth analysis of COAD/READ—the largest dataset within the resource, illustrating its power in elucidating methylation-mediated regulatory mechanisms underlying tumorigenesis (Fig. 4). Specifically, we identified aberrant methylation patterns in enhancers and CGI promoters of key cancer-related genes, such as *TCF4* and *MGAT5*, and demonstrated their potential as prognostic and diagnostic biomarkers. These findings highlight the value of CMAtlas in identifying clinically relevant epigenetic alterations and in providing mechanistic insights into cancer progression.

To further enhance CMAtlas, our future efforts will focus on several key areas. First, we will continuously expand the coverage and resolution of this platform. A major priority is to continuously monitor and systematically incorporate new, high-quality WGBS datasets, especially for cancer types where this data type is currently limited. In parallel, we will prioritize the acquisition of additional scMeth-seq data to better capture tumor epigenetic heterogeneity and cell type-specific methylation patterns. Second, we intend to develop more user-friendly analysis modules, such as tools for comparing methylation profiles across user-defined gene sets or genomic elements, and for exploring correlations between somatic mutations and methylation alterations. Third, we aim to incorporate machine learning and artificial intelligence approaches to predict cancer subtypes, survival outcomes, and therapeutic responses based on DNA methylation patterns, thereby augmenting the predictive utility of CMAtlas.

In conclusion, CMAtlas provides a comprehensive and user-friendly platform for exploring the role of DNA methylation in tumorigenesis. We anticipate that CMAtlas will serve as a valuable resource for researchers seeking to decipher the epigenetic basis of cancer and to develop promising diagnostic and therapeutic strategies.

## Supplementary Material

btag022_Supplementary_Data
