# Supplementary material for: CMAtlas: a comprehensive DNA methylation atlas for exploring epigenetic alterations in 34 human cancer types
Source: Bioinformatics. 2026 Jan 14;42(2):btag022. doi: 10.1093/bioinformatics/btag022 (PMC12881830; doi:10.1093/bioinformatics/btag022)
Supplement: btag022_Supplementary_Data [file btag022_supplementary_data.docx]

**Supplementary Materials**

**Supplementary Methods**

Analysis of array data

Methylation levels were quantified as beta values by SeSAMe (version 1.20) (Zhou et al., 2018), which were calculated as Meth/(UnMeth + Meth), where ‘Meth’ and ‘UnMeth’ refer to the methylated and unmethylated probe intensities, respectively. To ensure data quality and minimize false positive results, stringent probe filtering was applied as follows: (i) removal of probes overlapping with single nucleotide polymorphisms; (ii) exclusion of probes flagged as unreliable by the manufacturer; (iii) filtering out probes included in the ENCODE blacklist regions; and (iv) elimination of probes located on sex chromosomes and mitochondrial DNA. Principal component analysis (PCA) of DNA methylation patterns for genomic elements revealed low batch effects across different datasets, with clear separation of samples by cancer type, highlighting cancer-type-specific methylation patterns. This supports the validity of detecting cancer-type specific DM features for downstream analyses based on array data (**Fig. S2A-B**) .

Analysis of RRBS data

For tissue RRBS raw data, adapter sequences and inline barcodes were trimmed using Trim Galore (version 0.6.10). The cleaned reads were then mapped to the hg19 human genome using BSMAP (version 2.90)(Xi and Li, 2009), with the options “-q 20 -f 5 -r 0 -v 0.05 -s 16 -S 1”. CpG methylation calling was performed using MethylDackel (version 0.6.1) under the setting “­­minDepth 10”. Finally, all methylation coordinates were converted from hg19 to hg38 using the UCSC LiftOver tool (Genovese et al., 2024).

For cell line RRBS data, we adopted the processing pipeline described in the original publication. Briefly, raw sequencing reads were quality-trimmed using Trim Galore (version 0.6.10) and aligned to the hg38 genome with Bismark (version 0.24.2) (Krueger and Andrews, 2011). Uniquely mapped reads were extracted and sorted using SAMtools (version 1.13) (Li et al., 2009). Genome-wide CpG site methylation levels were calculated using methylKit (version 1.28.0) (Akalin et al., 2012) with the parameters: “read.context = "CpG", mincov = 10, minqual = 20”. Consistent with the array data analysis, PCA demonstrated that cancer-type-specific methylation patterns are also largely conserved across different RRBS datasets of the same cancer type. (**Fig. S2C-E**).

Analysis of WGBS data

Raw WGBS datasets underwent quality control and preprocessing using Trim Galore (version 0.6.10) to remove adaptors and low-quality bases. The trimmed reads were then aligned to the hg38 reference genome using Bismark (version 0.24.2) (Krueger and Andrews, 2011) with default parameters. PCR duplicates were removed using the Deduplicate_bismark command. Subsequently, Bismark was employed to extract genome-wide CpG site methylation levels. For processed datasets previously aligned to the hg19 reference genome, genomic coordinates were converted to the hg38 assembly using the UCSC liftOver tool (Genovese et al., 2024). To ensure the reliability of subsequent analyses, CpG sites with a sequencing depth of fewer than 10 reads were excluded from all downstream computations.

Analysis of scMeth-seq data

The preprocessing BedGraph files of prostate adenocarcinoma (PRAD) were first converted from the hg19 to the hg38 genome assembly using the UCSC LiftOver tool (Genovese et al., 2024). CpG sites overlapping ENCODE blacklist regions were subsequently filtered out. To ensure the robustness of downstream differential methylation analysis, only patients with a minimum of 10 cells were included.

Analysis of cfDNA methylation data

cfDNA methylation data collected in CMAtlas were derived from multiple profiling technologies, including Infinium EPIC arrays, RRBS, and WGBS. The same pre-processing pipeline applied to tissue data was adopted for cfDNA data from the corresponding sequencing technologies. Given the limited number of reliably detectable CpG sites in cfDNA sequencing data, we retained CpG sites with ≥3× sequencing coverage for downstream analysis.

Multi-omic annotations of differential methylated features

CMAtlas provided multi-omics annotations for DM features. For gene expression, all DM features were annotated with the log₂(TPM + 1) values of their corresponding genes. For somatic mutations, DM features were annotated with any mutations located within their genomic regions. Additionally, ATAC-seq signals were extracted using the get_coverage function from megadepth (version 1.2.0) (Wilks et al., 2021) and subsequently assigned to DM features based on genomic coordinate overlap.

Identification of enriched biological functions and TFs

To investigate the biological implications of the DMEs and DMRs, we first performed functional enrichment analysis. Gene Ontology (GO), KEGG pathway, and Reactome pathway analyses were conducted using clusterProfiler (version 4.10.1) (Wu et al., 2021) and ReactomePA (version 1.46.0) (Yu and He, 2016), respectively. Subsequently, TF binding motif enrichment within DMEs/DMRs was analyzed using the findMotifsGenome.pl function in Homer. Human core TF binding models were derived from the HOCOMOCO database (v11) (Kulakovskiy et al., 2018). All enrichment analyses employed the hypergeometric test with Benjamini–Hochberg multiple testing correction, and significantly enriched terms were defined as those with a FDR < 0.05.

**Supplementary Figures**

**
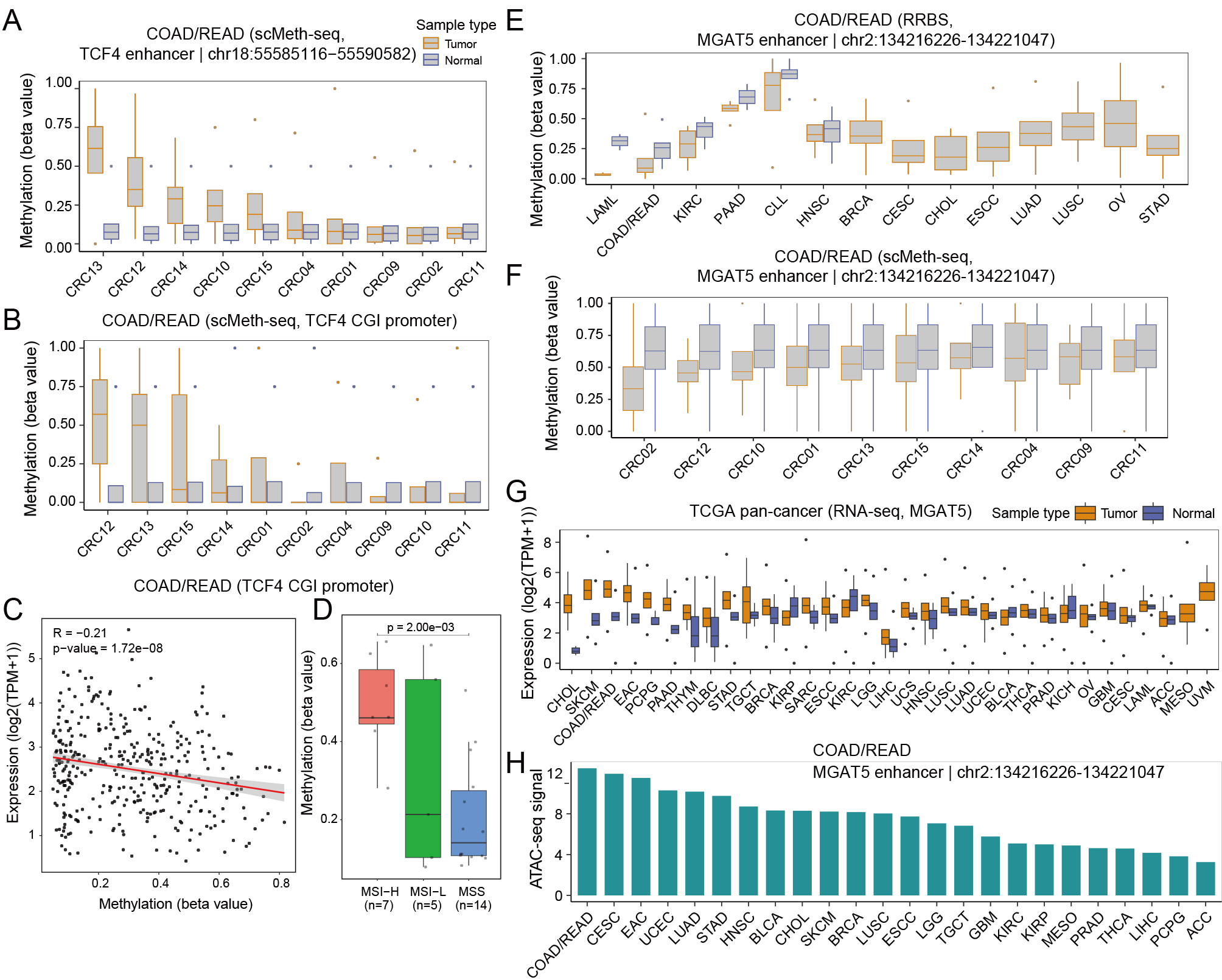
**

**Fig. S1.** **Application of CMAtlas in COAD/READ analysis.** **(A)** Methylation profile of each COAD/READ patient profiled by scMeth-seq for hypermethylated *TCF4* enhancer. **(B)** Methylation profile of each COAD/READ patient profiled by scMeth-seq for *TCF4* CGI promoter. **(C)** Correlation between methylation level of the *TCF4* CGI promoter and *TCF4* expression using Pearson correlation. **(D)** Comparison of COAD/READ molecular subtypes based on methylation levels of the *TCF4* CGI promoter. **(E)** Pan-cancer DNA methylation profile from RRBS data for *MGAT5* enhancer. **(F)** Methylation profile of each COAD/READ patient profiled by scMeth-seq for *MGAT5* enhancer. **(G)** Pan-cancer gene expression profile of the *MGAT5* based on TCGA RNA-seq data. **(H)** Pan-cancer chromatin accessibility profile from TCGA ATAC-seq for the hypermethylated *MGAT5* enhancer.


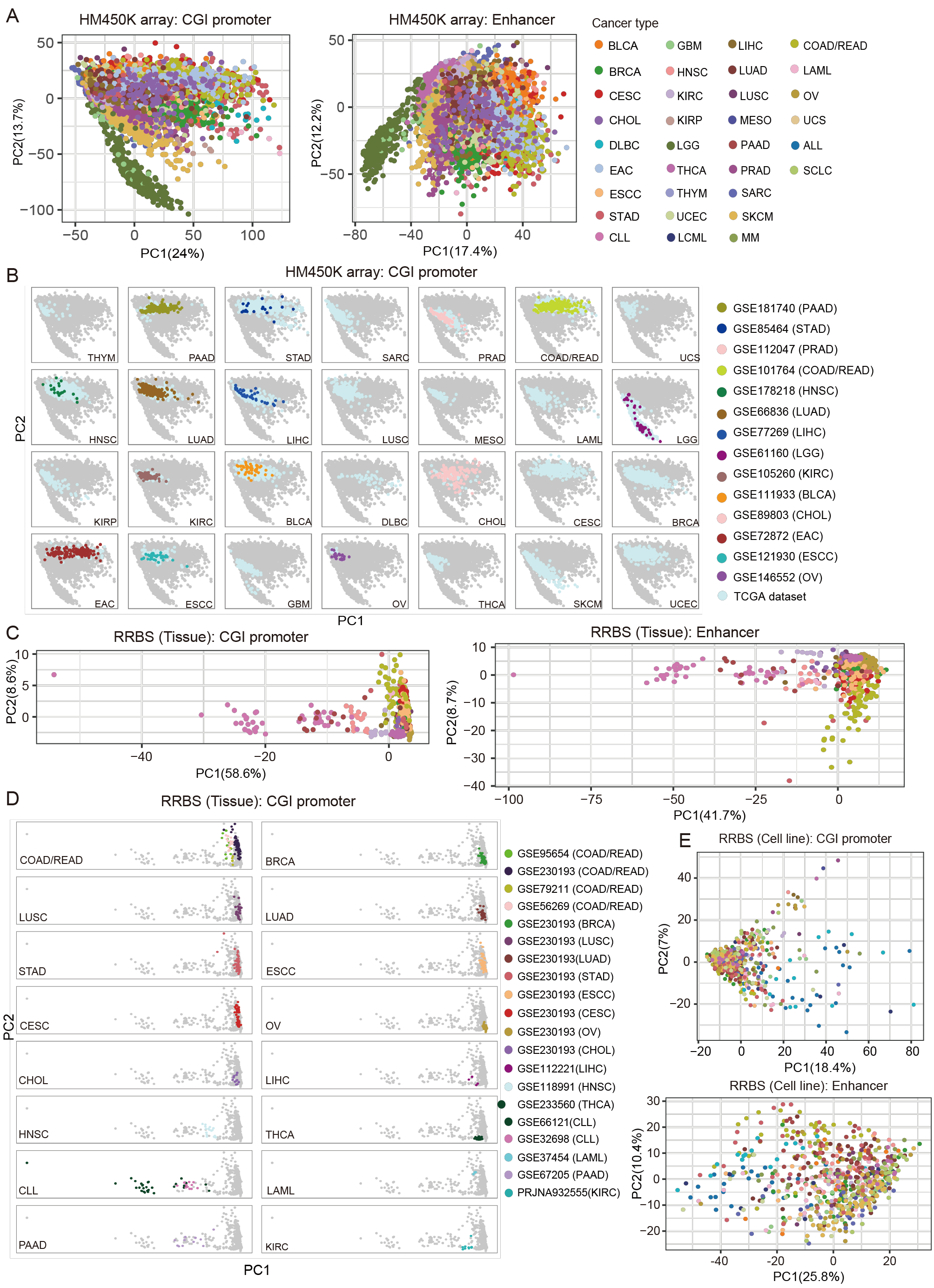


**Fig. S2. Principal component analyses demonstrate cancer-type-specific methylation patterns**. **(A)** PCA of DNA methylation patterns in CGI promoter and enhancer regions across 28 cancer types using HM450K array data. **(B)**PCA of CGI promoter methylation profiles across 28 cancer types using HM450K array data, with datasets color-coded by cancer type. **(C)**PCA of methylation patterns for CGI promoter and enhancer regions across 16 cancer types using tissue-derived RRBS data; **(D)**PCA of CGI promoter methylation across 16 cancer types based on tissue RRBS data, with datasets color-coded by cancer type. **(E)** PCA of DNA methylation landscapes in CGI promoter and enhancer regions across 25 cancer types, derived from RRBS data of tumor cell lines.

**References**

Akalin, A., Kormaksson, M., Li, S., Garrett-Bakelman, F.E., Figueroa, M.E., Melnick, A., Mason, C.E., 2012. methylKit: a comprehensive R package for the analysis of genome-wide DNA methylation profiles. Genome Biol 13(10), R87. <https://doi.org/10.1186/gb-2012-13-10-r87>.

Genovese, G., Rockweiler, N.B., Gorman, B.R., Bigdeli, T.B., Pato, M.T., Pato, C.N., Ichihara, K., McCarroll, S.A., 2024. BCFtools/liftover: an accurate and comprehensive tool to convert genetic variants across genome assemblies. Bioinformatics 40(2). <https://doi.org/10.1093/bioinformatics/btae038>.

Krueger, F., Andrews, S.R., 2011. Bismark: a flexible aligner and methylation caller for Bisulfite-Seq applications. Bioinformatics 27(11), 1571-1572. <https://doi.org/10.1093/bioinformatics/btr167>.

Kulakovskiy, I.V., Vorontsov, I.E., Yevshin, I.S., Sharipov, R.N., Fedorova, A.D., Rumynskiy, E.I., Medvedeva, Y.A., Magana-Mora, A., Bajic, V.B., Papatsenko, D.A., Kolpakov, F.A., Makeev, V.J., 2018. HOCOMOCO: towards a complete collection of transcription factor binding models for human and mouse via large-scale ChIP-Seq analysis. Nucleic Acids Res 46(D1), D252-D259. <https://doi.org/10.1093/nar/gkx1106>.

Li, H., Handsaker, B., Wysoker, A., Fennell, T., Ruan, J., Homer, N., Marth, G., Abecasis, G., Durbin, R., Genome Project Data Processing, S., 2009. The Sequence Alignment/Map format and SAMtools. Bioinformatics 25(16), 2078-2079. <https://doi.org/10.1093/bioinformatics/btp352>.

Wilks, C., Ahmed, O., Baker, D.N., Zhang, D., Collado-Torres, L., Langmead, B., 2021. Megadepth: efficient coverage quantification for BigWigs and BAMs. Bioinformatics 37(18), 3014-3016. <https://doi.org/10.1093/bioinformatics/btab152>.

Wu, T., Hu, E., Xu, S., Chen, M., Guo, P., Dai, Z., Feng, T., Zhou, L., Tang, W., Zhan, L., Fu, X., Liu, S., Bo, X., Yu, G., 2021. clusterProfiler 4.0: A universal enrichment tool for interpreting omics data. Innovation (Camb) 2(3), 100141. <https://doi.org/10.1016/j.xinn.2021.100141>.

Xi, Y., Li, W., 2009. BSMAP: whole genome bisulfite sequence MAPping program. BMC Bioinformatics 10, 232. <https://doi.org/10.1186/1471-2105-10-232>.

Yu, G., He, Q.Y., 2016. ReactomePA: an R/Bioconductor package for reactome pathway analysis and visualization. Mol Biosyst 12(2), 477-479. <https://doi.org/10.1039/c5mb00663e>.

Zhou, W., Triche, T.J., Jr., Laird, P.W., Shen, H., 2018. SeSAMe: reducing artifactual detection of DNA methylation by Infinium BeadChips in genomic deletions. Nucleic Acids Res 46(20), e123. <https://doi.org/10.1093/nar/gky691>.
